# Explicit Agency in Patients with Cervical Dystonia: Altered Recognition of Temporal Discrepancies between Motor Actions and Their Feedback

**DOI:** 10.1371/journal.pone.0162191

**Published:** 2016-08-30

**Authors:** Cécile Delorme, Emmanuel Roze, David Grabli, Jean-Michel Mayer, Bertrand Degos, Marie Vidailhet, Yulia Worbe

**Affiliations:** 1 UMR S 975, CNRS UMR 7225, ICM, Sorbonne Universités, UPMC University Paris 06, Paris, France; 2 Department of Neurology, Groupe Hospitalier Pitié-Salpêtrière, Assistance Publique-Hôpitaux de Paris, 47–83 boulevard de l’Hôpital, Paris, France; 3 French Reference Centre for Gilles de la Tourette Syndrome, Groupe Hospitalier Pitié-Salpêtrière, 47–83 boulevard de l’Hôpital, Paris, France; University of Florida, UNITED STATES

## Abstract

**Background:**

Abnormalities in the cognitive processing of movement have been demonstrated in patients with dystonia. The sense of agency, which is the experience of initiating and controlling one’s own actions, has never before been studied in these patients.

**Objectives:**

We investigated whether the sense of agency is altered in patients with cervical dystonia.

**Methods:**

We used an explicit metacognitive agency task in which participants had to catch targets with a cursor by moving a computer’s mouse. The task included several conditions in which the control over the cursor could be disrupted by adding a spatial or a temporal discrepancy between the mouse and the cursor’s movements. Participants had to acknowledge these discrepancies and reflect them in metacognitive judgements of agency.

**Results:**

Twenty cervical dystonia patients and 20 matched controls were included in the study. Despite performing equally well as the matched controls, cervical dystonia patients did not fully recognize alterations of agency when a temporal lag was added between their movement and the visual feedback. Moreover, they relied predominantly on their perceived performance to provide judgements of agency and less on their objective degree of controls. There was no correlation between agency scores and clinical severity of dystonia measured by the Toronto Western Spasmodic Torticollis Rating Scale.

**Conclusion:**

We demonstrated an abnormal processing of agency in cervical dystonia patients, even for motor actions not affected by dystonia. The exact contribution of abnormal agency to dystonia pathophysiology remains to be clarified.

## Introduction

Dystonia is characterized by sustained or intermittent involuntary muscle contractions causing abnormal, repetitive movements, or abnormal postures [1]. Primary dysfunctions associated with dystonia include loss of inhibition at various levels of the central nervous system, alteration of synaptic plasticity, and sensory dysfunction [2]. Dystonia is increasingly conceptualized as a disorder of motor organization, programming and execution [3], and sensorimotor integration (i.e., the ability to use sensory information to guide motor program execution) [4]. Indeed, experimental studies have noted alterations of spatial [5] and temporal discrimination [6] of sensory stimuli in a variety of focal and generalized dystonia syndromes. Moreover, kinesthetic [7] and proprioceptive [8] impairments have been demonstrated.

Beyond motor organization and execution, abnormal cognitive processing of movement has been shown in dystonia, including alterations of movement and body representation [9], mental rotation of body parts [10], and temporal processing of movement [11].

The sense of agency is part of the cognitive processing of movement. It refers to the experience of initiating and controlling one’s own actions [12]. Agency is a supra-modal, high-order phenomenon integrating several functional heterogeneous levels [13]. The low-order level is an implicit process related to the comparator model of action control. If the efference copy of a motor plan matches the actual sensory effects of the action, the action is perceived as self-generated and the person experiences a “feeling of agency” [14]. The sense of agency also encompasses a higher-order, metacognitive, self-reflective dimension, the explicit “judgement of agency” (JoA) formed by synthetizing information of “feeling of agency” in light of other cues such as perceived performance and personal beliefs (i.e., understanding of the world based on prior experiences) [15].

The sense of agency has never before been investigated in patients with dystonia. We decided to test the hypothesis that patients with cervical dystonia (CD) have alterations of the sense of agency. To this aim, we administered an explicit metacognitive agency task to CD patients and healthy controls. This paradigm involves actions of the upper limbs. Since a significant subset of patients with CD have associated symptoms in the shoulders and the upper limbs [16], we only included patients with dystonia restricted to the neck muscles, with no impairment in the movement of their upper limbs. This task evaluated whether subjects were able to acknowledge discrepancies between motor actions and their visual feedback and to reflect these discrepancies in metacognitive JoA [17].

## Materials and Methods

### Participants

Participants were included in the protocol from January through October 2015. Patients were recruited through the Movement Disorders Clinics of the Pitié-Salpêtrière Hospital in Paris. Healthy volunteers were recruited through the resource for biomedical research volunteers and university-based advertisements. Patients and controls provided written consent to participate in the study. The experimental procedure was approved by the ethics committee of the Pitié-Salpêtrière Hospital (97/12).

The inclusion criteria for the patients included: i) a diagnosis of isolated adult-onset CD; ii) the presence of the disease for at least one year; iii) no botulinum toxin injections for at least three months prior to the study; and iv) normal or corrected-to-normal vision.

The exclusion criteria included any neurological history except for dystonia, a history of dopamine antagonist treatment, secondary dystonia, dystonia in the upper limbs, or the inability to maintain a straight gaze. The clinical severity of dystonia was assessed using the Toronto Western Spasmodic Torticollis Rating Scale (TWSTRS) [18] by a movement disorder expert (ER) who was blinded to the results of the task.

Patients and healthy volunteers were matched according to age, gender, and educational level. All of the patients and controls were right-handed. The inclusion criteria for the controls were an age over 18 years and normal or corrected-to-normal vision. The exclusion criteria included any neurological disorder and the ingestion of any type of medication, except contraceptive pills for women. The demographic and clinical data of the participants are listed in **Table 1**.

**Table 1:** Demographic data of subjects included in the study.

	Cervical dystonia (n = 19)	Controls (n = 20)	p
**Age**	48.53 ± 2.14	45.20 ± 3.26	0.400 (a)
**Gender (F:M)**	16 : 3	14 : 6	0.292 (b)
**Years of study**	13.32 ± 0.61	14 .55 ± 0.44	0.109 (a)
**Disease duration (years)**	8.37 ± 1.15	NA	
**TWSTRS**	12.2 ± 2.96	NA	

Reported as Mean ± SEM.

(a): two samples t-test

(b): χ2 test.

TWSTRS = Toronto Western Torticollis Rating Scale

### Agency Task

A detailed description of the task can be found elsewhere [19].

**Fig 1:**
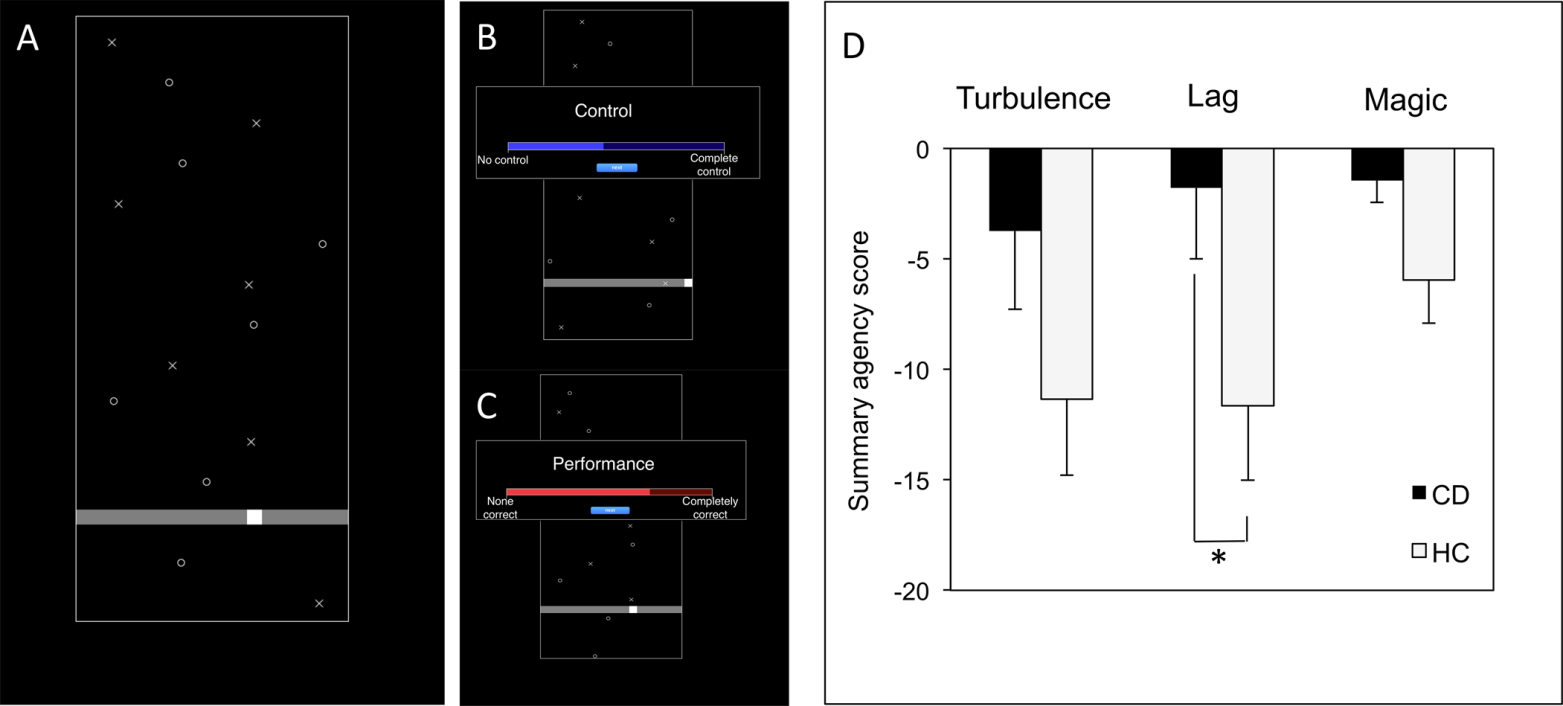
Experimental task and main results about here. **A.** Game phase of the task. The participant moves the mouse to move the cursor in order to touch the targets (Xs) and avoid the distractors (Os). **B, C.** Judgement phase of the task. The participant clicks on the blue bar to report his or her judgement of control over the cursor’s movement (B). Likewise, the participant clicks on the red bar to report his or her judgement of goodness of performance (C). **D.** Results of the summary agency score for every experimental condition. CD = cervical dystonia patients. HC = healthy controls.

Briefly, the subjects were seated in front of a computer screen with the computer mouse in their dominant hand. Targets (Xs) and distractors (Os) were spread across a black window and scrolled down at a regular speed. A white cursor could be moved along a horizontal track by making right-left movements with the computer mouse. The aim of the task was to touch the targets with the cursor and to avoid the distractors. Hitting the targets or distractors would make them disappear; otherwise, they would continue to scroll down (Fig 1A).

Hitting a target would cause a “beep” sound; hitting a distractor would cause a “boop” sound. The task consisted of 24 game sessions, each lasting 15 seconds; there was one practice session.

At the end of each game session, the participant was asked to provide a judgement of control or JoA, i.e., to what extent he or she experienced control over the cursor’s movement. The participant was also asked to provide judgement of performance (JoP), i.e., to what extent the participant estimated he or she succeeded at hitting the targets and avoiding the distractors. The judgements were reported by clicking on visual analogue scales; the right end of the bar corresponded to total control or perfect performance, and the left end of the bar corresponded to no control or poor performance. Judgements were assigned a proportional value between 0 and 100 (Fig 1B and 1C).

The sessions were divided in four game conditions. There were six sessions for each condition in a randomized order. In the “Control” condition, there was a full spatial and temporal congruence between the mouse and cursor’s movements. In the “Turbulence” condition, programmed random noise was added to the cursor’s movements. This noise introduced spatial and temporal incongruence between the mouse and cursor’s movements and disrupted the participant’s objective control over the cursor. In the “Lag” condition, a 500-ms temporal delay was added between the mouse and cursor’s movements. Finally, in the “Magic” condition, the targets would disappear if they were within 10 pixels of the cursor, even if they were not actually hit. This manipulation inflated the participant’s performance without modifying the objective control compared with the Control condition.

### Outcome Measures

We tested whether participants were able to acknowledge, in the three experimental game conditions (Turbulence, Lag, and Magic), discrepancies between their motor actions and the feedback they obtained from the cursor’s movements. For this purpose, we computed in each experimental game condition a summary agency score according to the following equation: deltaJo_E_ = (JoP_C−_JoA_C_)–(JoPE−JoA_E_), where E refers to the experimental condition studied and C refers to the Control condition. This score limited scaling effects since it studied perceived control relative to perceived performance. In healthy subjects, this score was previously shown to be negative in the three experimental conditions since participants realized that their performance was not entirely within their own control [19].

The secondary outcome measures included performance in each condition, assessed as the mean number of target hits divided by the total number of targets, and the mean amount of cursor movement during the game sessions.

### Statistical Analysis

We performed the statistical analyses using the Statistical Package for Social Science (SPSS) version 22.0 (SPSS Inc. Chicago, IL, USA). Prior to the final analysis, all of the variables were tested for a Gaussian distribution (Shapiro-Wilk test, p>0.05), with a log or square root transformation, if appropriate. Outlier data (>3 standard deviations above the group mean) were removed from the final analysis. The demographic data were analyzed using two-sample *t*-tests and chi-squared tests, where appropriate.

The between-groups comparisons of the primary outcome measures (summary agency scores, performance, and movement) of the task was performed using multivariate ANOVA with group as an independent factor and outcome measure in each task condition as a dependant variable. Prior to the multivariate ANOVA, the summary agency scores were tested using a one-sample *t*-test in each group and each condition as a baseline statistical analysis; this score ought to be significantly smaller than 0 [19].

Furthermore, using multiple regression analysis, we addressed the question of specific factors that contributed independently to JoA in each group. To this end, we first dummy-coded the condition as being of a “normal control” (value = 0, for the Control and Magic conditions), or of an “altered control” (value = 1, for the Turbulence and Lag conditions). Then, we performed regression analysis using JoA as a dependent factor and JoP and condition type as independent variables.

The results are shown as standardized regression coefficients beta, which are measures of how many standards deviation of the dependent variable changes per standard deviation change in the explanatory variable. Finally, to evaluate whether the clinical parameters (TWSTRS score, disease duration) impacted the outcome task measures, we performed regression analysis after 5000 bootstrap iterations with the Mersenne-Twister generator of random numbers.

## Results

### Subjects

Twenty CD patients and 20 controls were enrolled in the study. One CD patient was excluded from the final analysis since she reported taking a dopamine-blocking medication after being included in the study.

Among the remaining 19 CD patients, 17 were treated with botulinum toxin type A; the mean interval since the last injection was 4.27 months (range: 3–12 months). Two CD patients were naïve from botulinum toxin treatment, and five patients had head tremor.

### Summary Agency Scores

Prior to the analysis of the summary agency scores, we compared the raw JoA and JoP scores in the two groups across task conditions using one-way ANOVA. This analysis revealed no statistically significant difference between the two groups in any task condition (see Table 2 for the statistical report).

**Table 2:** Outcomes measures in each condition.

Measure	CD	HC	P
**Control**			
Performance (% hits)	70.87 ± 2.71	73.51 ± 3.08	0.689
JoA (%)	67.27 ± 4.04	73.15 ± 3.60	0.283
JoP (%)	65.68 ± 3.97	65.73 ± 3.24	0.991
Movement (mm)	3142.23 ± 243.72	3230.16 ± 164.28	0.764
**Turbulence**			
Performance (% hits)	41.73 ± 1.22	39.97 ± 1.40	0.379
JoA (%)	35.26 ± 4.31	31.22 ± 2.72	0.427
JoP (%)	37.53 ± 4.30	35.14 ± 2.74	0.640
deltaJo	- 3.74 ± 3.55	- 11.34 ± 3.45	0.108
Movement (mm)	3796.81 ± 330.22	3694.24 ± 232.66	0.799
**Lag**			
Performance (% hits)	22.31 ± 1.05	23.52 ± 1.28	0.506
JoA (%)	24.07 ± 4.35	19.78 ± 2.23	0.379
JoP (%)	24.39 ± 4.63	24.01 ± 2.63	0.943
deltaJo	- 1.79 ± 3.22	- 11.65 ± 3.36	0.033
Movement	6040.10 ± 692.69	5747.47 ± 571.95	754
**Magic**			
Performance (% hits)	91.65 ± 2.13	93.77 ± 1.88	0.461
JoA (%)	74.52 ± 4.09	83.22 ± 2.69	0.081
JoP (%)	77.18 ± 3.99	81.77 ± 3.21	0.372
deltaJo	- 1.47 ± 0.97	- 5.97 ± 1.94	0.053
Movement	3164.53 ± 182.21	3154.93 ± 190.56	0.971

Results are shown as mean ± SEM.

Abbreviations: CD = cervical dystonia patients. HC = healthy controls. JoA = judgment of agency. JoP = judgment of performance. deltaJo = summary agency score.

Our one-sample *t*-test analyses revealed that all of the summary scores were significantly smaller than 0 in the controls (Turbulence: t = -3.289, p = 0.004; Lag: t = -3.463, p = 0.003; Magic: t = -3.072, p = 0.006) as expected, but not in CD patients (Turbulence: t = -1.052, p = 0.307; Lag: t = -0.556, p = 0.585; Magic: t = -1.520, p = 0.147).

Multivariate ANOVA analysis revealed an effect of group on the summary agency score in the Lag condition (F_1,38_ = 4.888, p = 0.033; η^2^ = 0.12); there was no difference in the Magic condition (F_1,38_ = 4.004, p = 0.053, η^2^ = 0.07) or the Turbulence condition (F_1,38_ = 2.721, p = 0.108, η^2^ = 0.10).

The summary score in the Lag condition of the task was higher in CD patients compared with the controls (Mean ± SEM: CD patients: -1.789 ± 3.215; Controls: -11.650 ± 3.364) (Fig 1D). The means and SEM are listed in Table 2.

### Performance

Using multivariate ANOVA analysis with groups as independent factors and performance on each task condition as dependent factors, we found no significant differences between the groups in terms of task performance: Control condition (F_1,38_ = 0.163, p = 0.689; η^2^ = 0.05), Lag condition (F_1,38_ = 0.451, p = 0.506; η^2^ = 0.01), Magic (F_1,38_ = 0.556, p = 0.461; η^2^ = 0.02), and Turbulence conditions (F_1,38_ = 0.795, p = 0.379; η^2^ = 0.02). Means and SEM are provided in Table 2. The co-occurrence of head tremor had no effect on performance (Control condition: p = 0.234, Turbulence condition: p = 0.791, Lag condition: p = 0.139, Magic condition: p = 0.514).

### Movement

Multivariate ANOVA analysis with groups as independent factors and the amount of movement in each task condition as dependent factors revealed no effect of group on the amount of movement in the different task conditions: Control condition (F_1,38_ = 0.091, p = 0.764; η^2^ = 0.002), Lag condition (F_1,38_ = 0.107, p = 0.754; η^2^ = 0.002, Magic (F_1,38_ = 0.001, p = 0.971; η^2^ = 0.000), and Turbulence conditions (F_1,38_ = 0.066, p = 0.799; η^2^ = 0.003). Means and SEM are provided in Table 2.

### Regression Analysis with Behavioral Scores

In both groups, we found a significant contribution of both JoP (CD patients: beta = 0.793, p<0.001; Controls: beta = 0.648, p<0.001) and condition type (CD patients: beta = -0.164, p<0.001; Controls: beta = -0.321, p<0.001) to JoA.

Partial regression tests revealed that the contribution of JoP was more important in CD patients than in controls (p = 0.032). There was no significant difference between groups for the contribution of condition type (p = 0.112).

### Regression Analysis with Clinical Scores

We performed a regression analysis with deltaJoLag as the dependent variable and disease duration and disease severity assessed by the TWSTRS score as independent variables. There was no contribution of disease duration (p = 0.566, SE = 1.262) or TWSTRS score (p = 0.512, SE = 1.018) to deltaJoLag.

## Discussion

We have demonstrated an alteration of the explicit processing of agency in patients with CD. These patients did not fully acknowledge loss of control over their actions when their objective visuo-motor control was artificially altered. In addition, these individuals were more prone to base their JoA on their perceived performance rather than on their objective level of control. It is noteworthy that CD patients exhibited normal performance in the task and that their amount of movement was equal to that of the controls.

### Altered Agency in Patients with CD

The task used in this study captures the metacognitive aspects of agency, as shown both in healthy subjects [17,19] and in patients with other disorders such as schizophrenia [20], autism spectrum disorders [21] or Gilles de la Tourette syndrome [22]. These previous studies showed that the summary agency score is a reliable measure for detecting perturbations in self-agency.

Here, we found significant differences between CD patients and controls in the Lag condition, characterized by disruption of temporal contiguity between the mouse and the cursor’s movements. Temporal contiguity is a major determinant to the sense of agency [23]. Healthy subjects are able to recognize a discrepancy between a motor action and its visual feedback when the temporal delay is more than 100–150ms [24].

The higher summary scores of CD patients compared with the controls indicated that CD patients did not explicitly recognize this discrepancy to the extent that healthy controls did. This result was substantiated by the regression analysis, which revealed that CD patients relied significantly more on their perceived performance than on the objective disruption of their control to provide JoA. There was only a trend for a higher agency score in the Turbulence condition, which may reflect the fact that this condition was less sensitive to subtle disruptions of perceived control. Nonetheless, the one-sample *t*-test showed that the summary score in this condition was not significantly different from 0, suggesting an alteration of agency even in this condition.

According to the optimal cue integration theory, different agency cues contribute to the sense of agency, and the relative influence of these cues is dependent on their reliability [25]. The results of the Lag and Turbulence conditions suggest that the mechanisms of detecting these discrepancies are abnormal in CD patients. Specifically, altered detection of a temporal discrepancy may be related to the known abnormalities in temporal processing in patients with dystonia [6,11]. Moreover, the results of the regression analysis suggest a decreased reliance on sensorimotor information and comparative processes and a relatively increased reliance on exteroceptive cues to form JoA.

The differences between the two groups in the agency measures could also be due to higher-order deficits of metacognition and self-reflection in patients with CD that impair their ability to produce metacognitive judgements of performance and agency. The nature of the task did not allow us to distinguish deficits in discrepancy recognition and metacognitive alterations.

Although our study did not address the neural bases of altered sense of agency in CD patients, our findings raise some interesting speculative hypotheses. For instance, dysfunction of the inferior parietal cortex is a plausible culprit for the perturbations of agency in CD patients. The inferior parietal cortex is involved in high-order sensory processing by integrating information from visuospatial perception, body scheme, and proprioception [26]. Multimodal integration of these multiple sources of information is used for movement preparation [27]. The inferior parietal cortex, and particularly the temporo-parietal junction and angular gyrus, are key regions implicated in sense of agency and movement awareness [28,29]. A previous fMRI study using the same task showed that the awareness of discrepancies between planned and actual movements was associated with increased activity in the temporo-parietal junction [30]. Patients with CD have an altered mental rotation of body parts, a task considered to rely on egocentric spatial perception and involving the parietal cortex [10]. Cerebellar dysfunction may contribute to agency impairments in CD patients. The cerebellum is involved in spatiotemporal predictions and comparative processes [31,32]. The role of the cerebellum in dystonia is now widely recognized [33]. Cervical dystonia patients exhibit abnormalities in motor timing occurring over timescales of milliseconds; these abnormalities are known to rely on cerebellar function [34]. Therefore, cerebellar dysfunction may have contributed to the abnormal responses of patients in the Lag condition.

### Clinical Relevance

One important question is whether abnormalities in the sense of agency are involved in dystonia pathophysiology and are associated with a primary dysfunction of the motor system.

We did not find any significant relation between clinical data and agency scores in the task. Similarly, previous studies of sensory dysfunction and alterations of motor representation in dystonia found no correlation with clinical parameters. Moreover, these abnormalities often involved unaffected body parts and even unaffected relatives of patients with dystonia, suggesting that these abnormalities might reflect an endophenotype [35,36].

Additional studies are necessary to clarify the link between altered sense of agency and dystonia.

### Limitations

Our work has several limitations. First, the selection of outpatients from a dystonia reference center may have induced a selection bias since these patients may not be representative of the CD population as a whole. However, we selected consecutive patients with a wide variety of inclusion criteria. Second, the fact that the majority of patients were treated with botulinum toxin may have influenced our results. Although patients were seen at the end of their efficacy interval (with a mean interval of 4.27 months after the last injection), botulinum toxin has been shown to induce remote central effects, which could possibly have impacted the processing of agency [37].

We did not control for the presence of psychiatric comorbidities in our population of patients. A higher frequency of psychiatric disorders such as depression had been reported in CD patients [38]. Both depression and attention biases related to pain or abnormal posture may impact the sense of agency. However, the use of a summary score as our primary measure of agency may limit this bias since it avoids scaling effects and assesses judgments relative to the patient’s own references. In addition, no effect on self-attribution (agency) has been shown in patients with major depressive episodes [39]. Similarly, in patients with functional movement disorders, a recent neuroimaging study demonstrated decreased functional connectivity in neuronal networks implicated in the sense of agency but no effect of depressive state on functional connectivity in this network [40].

Another potential caveat is that the presence of torticollis may have impacted the visuomotor capacity of our cohort of patients. However, we only included patients who were able to sit and keep their head straight. Moreover, any significant impairment in maintaining gaze straight would have impacted the performances of the patients, which was not the case.

Finally, the relatively small sample of patients limited the statistical power of our analyses, and some negative results, such as the correlation analysis findings, should be interpreted with caution.

## Conclusions

Using an explicit agency task, we showed that patients with dystonia have an abnormal metacognitive processing of agency for movements that are not affected by dystonia. These findings provide further insights into the cognitive processing of movement in these patients and add to the growing body of literature on abnormal motor representation and organization in dystonia. However, neural bases for these alterations and their clinical relevance in the expression of symptoms still remain to be clarified.
